# Enablers and inhibitors of the implementation of the Casalud Model, a Mexican innovative healthcare model for non-communicable disease prevention and control

**DOI:** 10.1186/s12961-016-0125-0

**Published:** 2016-07-22

**Authors:** Roberto Tapia-Conyer, Rodrigo Saucedo-Martinez, Ricardo Mujica-Rosales, Hector Gallardo-Rincon, Paola Abril Campos-Rivera, Evan Lee, Craig Waugh, Lucia Guajardo, Braulio Torres-Beltran, Ursula Quijano-Gonzalez, Lidia Soni-Gallardo

**Affiliations:** Fundación Carlos Slim, Plaza Carso, Lago Zurich 245, Torre Carso, Piso 20, Mexico City, 11529 Mexico; Eli Lilly and Company, Mexico City, Mexicoᅟ; C230 Consultores, Mexico City, Mexico

**Keywords:** Regional health planning, Health plan implementation, Health policy, Chronic disease, Implementation, Barrier, Health intervention, Health technology

## Abstract

**Background:**

The Mexican healthcare system is under increasing strain due to the rising prevalence of non-communicable diseases (especially type 2 diabetes), mounting costs, and a reactive curative approach focused on treating existing diseases and their complications rather than preventing them. Casalud is a comprehensive primary healthcare model that enables proactive prevention and disease management throughout the continuum of care, using innovative technologies and a patient-centred approach.

**Methods:**

Data were collected over a 2-year period in eight primary health clinics (PHCs) in two states in central Mexico to identify and assess enablers and inhibitors of the implementation process of Casalud. We used mixed quantitative and qualitative data collection tools: surveys, in-depth interviews, and participant and non-participant observations. Transcripts and field notes were analyzed and coded using Framework Analysis, focusing on defining and describing enablers and inhibitors of the implementation process.

**Results:**

We identified seven recurring topics in the analyzed textual data. Four topics were categorized as enablers: political support for the Casalud model, alignment with current healthcare trends, ongoing technical improvements (to ease adoption and support), and capacity building. Three topics were categorized as inhibitors: administrative practices, health clinic human resources, and the lack of a shared vision of the model.

**Conclusions:**

Enablers are located at PHCs and across all levels of government, and include political support for, and the technological validity of, the model. The main inhibitor is the persistence of obsolete administrative practices at both state and PHC levels, which puts the administrative feasibility of the model’s implementation in jeopardy. Constructing a shared vision around the model could facilitate the implementation of Casalud as well as circumvent administrative inhibitors. In order to overcome PHC-level barriers, it is crucial to have an efficient and straightforward adaptation and updating process for technological tools. One of the key lessons learned from the implementation of the Casalud model is that a degree of uncertainty must be tolerated when quickly scaling up a healthcare intervention. Similar patient-centred technology-based models must remain open to change and be able to quickly adapt to changing circumstances.

**Electronic supplementary material:**

The online version of this article (doi:10.1186/s12961-016-0125-0) contains supplementary material, which is available to authorized users.

## Background

Mexico faces a critical epidemic of non-communicable diseases (NCDs). The prevalence of type 2 diabetes mellitus (T2D) has increased significantly, rising from 4.0% in 1993 to 9.2% in 2012 [1, 2]. Among non-communicable diseases, T2D represents one of the most pressing concerns for the Mexican healthcare system, although others are concomitantly placing a strain on the population. The prevalence of hypertension and overweight/obesity has also increased by 28.5% and 56.5%, respectively, between 1993 and 2012 [3, 4]. Tackling NCDs is not only a concern for Mexico; the United Nations has included the reduction of premature mortality from NCDs as one of their Sustainable Development Goals.

These growths in NCD prevalence and the associated increase in costs have put financial strain on the Mexican healthcare system. Total health expenditure, encompassing both public and private sectors, represented 6.1% of Mexico’s GDP in 2010–2014 [5]. Cost-pressure projections expect this number to increase by 2%, or possibly more, by 2030 [6]. The cost of medical attention for the principal complications of T2D accounted for 87% of the direct costs [3]. Moreover, the current Mexican healthcare system generally focuses on treatment of current illnesses rather than on primary or secondary prevention [7–9].

This situation calls for innovative approaches that improve the quality of healthcare while reducing total costs. Casalud is a comprehensive NCD care model based on the use of patient-centred technologies, and it may play an integral role in the transformation of the current healthcare approach. The model operates through a five-pillar intervention, with each pillar having specific technologies that allow its implementation in primary health clinics (PHCs).

The first pillar is the proactive prevention and detection of chronic diseases, and includes two tools: the MIDO™ Mobile Cart and the MIDO™ Backpack. Although community-based screening has proven to be useful in identifying new cases of chronic diseases, a systematic review has not found any one successful specific approach or setting [10]. Out of the need for evidence, and cognizant of the need for a stronger evidence base behind practical screening tools, the Carlos Slim Foundation (FCS) designed the MIDO Mobile Cart. The Mobile Cart is an all-in-one system used in the primary care setting that includes medical equipment to measure weight, height, waist circumference, blood pressure, and glucose on site. The MIDO Backpack (similar to those used in favelas in Brazil [11]) is a portable version of the MIDO system, and includes a tablet, glucometer, blood pressure cuff, and measuring tape to screen for diabetes, hypertension, and abdominal obesity. Making chronic disease screening portable has been shown to increase the number of detected individuals, and assure that previously-unreached populations are incorporated into the healthcare system (such as residents of rural areas [12]). Early detection of chronic diseases has been shown to save the healthcare system money [13], especially costs associated with the use of urgent care facilities [14].

SI-MIDO software accompanies the MIDO system, and is set up for screeners to capture patient-reported risk factors. SI-MIDO then processes the risk factors through an algorithm to assess disease-specific risk, allowing screeners to provide personalized lifestyle modifications to screened individuals. Community healthcare professionals can take MIDO Backpack, with its SI-MIDO-equipped tablet, on home or community visits, and then refer persons with probable T2D or hypertension to PHCs to confirm diagnosis and initiate treatment. Linkage to care after mobile screening is still a difficult area, as patients being lost to follow-up is an ongoing obstacle (and not just in Mexico [15]).

The second pillar is the management of chronic diseases based on evidence, and includes the Chronic Disease Information System (SIC for its initials in Spanish), and a Digital Portfolio for healthcare professionals (HCPs). SIC is a hybrid (online/offline) database where physicians can capture patient data on NCD care, thereby improving care quality by standardizing healthcare protocols and implementing continuous monitoring. SIC processes patient data through a series of algorithms to classify patient health and follow-up status. All the results of measurements and lab tests, as well as the SIC analyses, are accessible to the patient’s physician, who can then prescribe lifestyle changes and the most appropriate pharmacological treatment. The Digital Portfolio is also a tool for HCPs and is comprised of applications and reference materials, including health calculators to estimate body mass index as well as cardiovascular and other health risks, and a digital library with national clinical practice guidelines.

The third pillar, continuous monitoring of medication supply chain, is carried out through AbastoNET. AbastoNET is an online information system that standardizes metrics for stock management at PHCs. Pharmacists use AbastoNET to register supply levels of medicines and lab tests, as well as stockouts of specific medicines. This pillar is aligned with other initiatives in developing countries that have attempted to denounce and raise awareness at a government level about inefficient supply chains, such as the Stop Stockouts program in South Africa [16]. The Stop Stockouts program has come up against the same types of problems as AbastoNET, namely, that, while the campaigns have raised awareness, drug supply decisions are still centralized and made at a state or federal level [17].

The fourth pillar, capacity building through continuous medical education, is executed through the Online Interactive Platform for Health Education (PIEENSO in Spanish). PIEENSO is a hybrid (online/offline) platform that confers two degrees with academic endorsement from national and foreign universities. The first degree is a 110-hour Online Certificate, the goal of which is to update physicians’ knowledge about NCDs and current prevention, treatment, and management strategies. The second is a 40-hour practical course in which physicians solve real-life cases to test their knowledge in practical settings (called the Competencies Certification).

The fifth pillar is patient engagement and empowerment, the practical tools of which are still in the design phase. It is clear that patient engagement measures should include assessment of the knowledge, confidence and skills to prevent and manage chronic disease, plus tools to implement related behaviours [18]. One of the first pilots of a practical tool to promote these components of patient engagement included two different mobile applications that could be used with smartphones or through text messages. The apps help patients understand their health, begin to self-monitor and interpret their own results, and implement lifestyle changes; a specific app for patients with diabetes allows patients to schedule medicine and appointment reminders, input measurements like glucose and weight, and receive immediate personalized feedback and educational messages. However, due to the characteristics of the patient population – elderly, rural, and with limited access to and familiarity with technology – it was decided that a change of strategy was needed. The evidence on mobile health technologies is still limited [19], and the use of mobile health in and of itself may not lead to patient engagement [20]. This is why now, more interactive and in-person tools and activities are currently being piloted in PHCs nationwide that will be more appropriate to the patient population.

Casalud began its pilot phase in 2009 in seven Mexican states [5]. After obtaining positive preliminary results, the FCS and the Mexican Ministry of Health formed an alliance in December 2012 to implement Casalud in PHCs nationwide, and to incorporate the care model as a component of the National Strategy for the Prevention and Control of Pre-obesity, Obesity and Diabetes.

As of 2016, Casalud has been implemented in 130 PHCs in 25 out of the 32 Mexican states through a public–private partnership between FCS and federal and state governments [5]. If a PHC implements all components of the Casalud intervention, it is eligible to join the Networks of Excellence of the National Strategy for the Prevention and Control of Pre-obesity, Obesity and Diabetes (hereinafter referred to as “Networks of Excellence”).

The entire model is developed and financed through the FCS, which means that neither the public healthcare system nor its users/beneficiaries incur any additional costs. Measurement and evaluation of the pilots was funded through the Lilly NCD Partnership (to help build the evidence base for patient-centred NCD interventions) and implemented by C230 Consultores in Mexico.

## Methods

This paper focuses on finding and assessing relevant enablers (elements that allow and promote the rapid and successful implementation of an intervention) and inhibitors (elements that might impede or hinder said implementation process) [21–24]. In identifying enablers and inhibitors in the implementation of the Casalud model, we follow the literature on community-based healthcare interventions that aim to measure and systematize seemingly subtle factors gathered from qualitative data [25, 26]. Even subtle ‘nudges’, when analyzed as a whole, may explain success or failure.

Taking into account the multidisciplinary composition of the research team and the diverse and ample amount of textual data available, the Framework Approach was selected as the most useful methodological tool for analysing, coding, and identifying elements and overarching themes [27, 28]. The Framework Approach has been gaining popularity as a means of analysing qualitative data in healthcare research because it can be used to manage qualitative data and undertake analysis systematically [29, 30].

The Framework Approach allows multiple researchers to code data in the same document, which strengthens analysis by reducing discrepancies and potentially biased interpretations. In our case, the Framework Approach allowed our research team to compare and agree on the coding of topics identified in interview transcripts, surveys and field notes. While coding, we used two operative definitions: enablers are elements that aid in the successful implementation of the Casalud model, whereas inhibitors are those elements that could impede or hinder the implementation of Casalud.

### Data collection

Data were collected over a 2-year period lasting from April 2013 to April 2015. A field research team, in collaboration with PHC directors and local authorities of the Mexican Ministry of Health, visited PHCs and conducted interviews and surveys. The research team used an array of mixed qualitative and quantitative methods for data collection: surveys, in-depth interviews, and participant and non-participant observations (described in more depth in Table 1). Participants included key figures (PHC personnel, including physicians, nurses and directors) at eight PHCs in two central Mexican states. PHCs were selected based on their participation in the Networks of Excellence.

**Table 1:** Data collection instruments

Method	Data	Observations
Surveys	37 PHC HCPs shadowed and interviewed over a 2-week period in April 2015	2 PHC directors
		16 Physicians
		18 Nurses
		1 MIDO Mobile Cart operator
	224 PHC HCP self-administered questionnaires	5 PHC directors
		38 Physicians
		41 Nurses
		15 Other health providers
		10 Social workers
		9 Other (operative and administration)
In-depth interviews	68 in-depth interviews conducted over a 2-year period (2013–2015)	8 PHC directors
		2 Local healthcare district heads
Participant and nonparticipant observation	Field notes gathered over a 2-year period (2013–2015)	8 PHCs in two Mexican states

PHCs, primary health clinics; HCP, Healthcare professionals

Surveys were designed to identify main HCP workplace tasks in addition to their views on enablers and inhibitors in the implementation process. A total of 224 self-administered mixed qualitative and quantitative questionnaires were distributed each year during the aforementioned 2-year period, to the PHC personnel working that year (for details, see Table 1). Additionally, a subset of 37 PHC workers were shadowed and interviewed during a 2-week period in April 2015, allowing researchers to corroborate and update the results obtained from surveys. The research team interviewed eight PHC directors and two local health department heads over the 2-year period. Interviews with directors were carried out every 3 months, resulting in 64 interviews. Local healthcare district heads were interviewed four times (once every 6 months), for a total of 68 interviews. An interview guide that favoured depth over structure was designed, including questions about potential enablers and inhibitors, as well as overall perception of the model, its tools and the quality of support services. The interview guide was constantly updated due to the ongoing nature of the data collection process. Finally, a field team collected data through both participant and non-participant direct observation over the 2-year period. The team focused on assessing the implementation of Casalud’s components, in particular, how PHC personnel used the T2D and other NCD detection tools. As a result, the field team produced a large quantity of field notes, which were systematized for analysis.

### Ongoing monitoring within the model for each tool

Each pillar has an ongoing monitoring system that allows for Casalud’s implementers to ensure that each component is being implemented with fidelity. Data were taken from each of the data sources described below for each component.

For the first pillar, containing the MIDO Mobile Cart and Backpack, each participating PHC has a screening goal to be met. This incentivizes the PHCs to capture screened individuals using the SI-MIDO software, which is then uploaded onto a public online Dashboard (*Tablero*) [31]. Information on the screening goals and progress of PHCs is broken down by screenings performed with the Mobile Cart and the Backpack, and is updated weekly.

The information taken from SIC, a component of the second pillar of evidence-based NCD care, is also publically available through its own Dashboard [32]. The most valuable information available through the SIC Dashboard is the percentage of patients with a certain condition that have corresponding laboratory test results. For example, as of June 2016, out of 961,733 patients with diabetes, only 20.0% had an A1c measurement; however, only 40.7% of patients with an A1c measurement had their A1c levels in control (below 7%). The SIC Dashboard allows a breakdown of this information by state, local and PHC level, empowering local authorities to base their health policy decisions on the areas of most need.

AbastoNET, the third pillar, is by nature an ongoing monitoring mechanism for PHCs to input medication supply and prescriptions filled for eight medications essential for NCD care. PHC directors can then use these metrics, broken down daily and monthly, to order new medications and to guide prescribing decisions. However, while these reports are important for understanding the internal inventory of PHCs, they are not shared with higher supply chain decision-making authorities.

To measure the implementation of the fourth pillar, ongoing training, the FCS uses PIEENSO’s internal database, which captures information on the characteristics of the participants, their baseline knowledge and improvement after each course. On a larger scale, the PIEENSO platform contributes to the National Strategy, specifically Objective 2.3 ‘Improving Skills and Capacity of Healthcare Personnel’. According to this objective, all institutions in the Mexican healthcare system must have formal training that is online, personalized, linked to continuing education, validated by academic organizations and monitored. The PIEENSO platform fulfils all of these requirements, and is an important tool to monitor the percentage of HCPs who have been trained.

## Results and discussion

We found seven recurrent topics in the analyzed textual data and categorized them into the two previously mentioned main types: enablers and inhibitors in the implementation of the Casalud model. We give detailed examples of each enabler and inhibitor linked to the tool(s) where it was most commonly referenced in the text. Additionally, and to ease interpretation, we have further divided our analysis according to the relevant pillar of Casalud (Table 2). Although certain elements were clear enablers or clear inhibitors across all tools or pillars of Casalud, some were found to be mixed enablers and inhibitors. Indeed, certain enablers for one tool proved to be inhibitors for others (as was the case for adaptation and technical support of the evidence-based disease management pillar).

**Table 2:** Enablers and inhibitors by pillar of Casalud

Pillar	Predominantly enablers					Predominantly inhibitors		
	Political support	Current healthcare trends	Availability of technology	Adaptation and technical support	Capacity building	Administrative practices	PHC human resources	Lack of shared vision
1. Proactive prevention and detection	Enabler	Enabler	Enabler/Mixed	Enabler/Mixed	Enabler	Inhibitor	Inhibitor	Inhibitor/Mixed
2. Evidence-based disease management	Enabler/Mixed	Enabler	Enabler	Inhibitor	Enabler	Inhibitor	Inhibitor	Inhibitor
3. Monitoring of medication supply chain	Mixed	Enabler	Enabler	NA	NA	Inhibitor	NA	Inhibitor
4. Capacity building through continuous education	Enabler	Enabler	Enabler	Enabler	Enabler	NA	Mixed	NA

Source: Designed by authors based on analyzed data

NA, Not enough information or does not apply; PHCs, primary health clinics

### Enablers

#### Political support for the Casalud model

The first enabler identified was that key federal officials in the health policy sector support the Casalud model, given that Casalud is a potential solution for primary and secondary NCD prevention issues in Mexico. The tools that enjoyed the most political support were the MIDO Mobile Cart and the PIEENSO platform, because they were closely aligned with the National Strategy for the Prevention and Control of Pre-obesity, Obesity and Diabetes. This strategy is an executive order, and is the main guiding document for the administration at the executive branch level regarding NCD awareness, prevention and care. The perception of key policymakers at a federal level is that the model can become an effective intervention for reducing burdens and costs to the Mexican healthcare system. This is shown by the role Casalud plays by setting best practices and by elevating the standard of care for NCDs. Casalud’s screening and detection activities help the National Strategy to meet its population coverage goals; and Casalud’s program of continued education for HCPs assists the National Strategy in meeting its professional training goals.

The FCS has had, and continues to have, important opportunities to engage key officials and lobby for innovative and preventative approaches to be included in primary healthcare policy. The FCS’s ability to effectively communicate with key policymakers allowed the message of Casalud’s comprehensive and preventive approach to permeate throughout the federal level of healthcare policy. However, there were certain sensitive and delicate subjects where political actors presented more resistance to the implementation of specific Casalud tools. The clearest example was during the monitoring of the medication supply chain, where Casalud’s involvement with a thorny issue could have put the implementation of AbastoNET at risk.

#### Relation to current healthcare trends

We found that most interviewed participants considered that Casalud’s implementation was facilitated by its consistency and harmonization with current research and practice in health policy. In fact, every tool evaluated was found to be aligned with international best practices and clinical practice guidelines, a key factor in receiving the abovementioned political support.

In particular, Casalud promotes innovative concepts and practices such as proactive prevention with early detection, evidence-based disease management throughout the continuum of care, patient-centred care, the use of innovative technologies in primary care settings, and capacity-building programs [8]. Casalud’s components are technology-based innovations designed to operationalize scientific and policy interventions. The implementation of the model is, thereby, a means to modify healthcare by using technology to implement evidence-based knowledge. For example, pre-diabetes, pre-hypertension or pre-obesity can be identified with the MIDO Mobile Cart or the MIDO Backpack in time for lifestyle changes; this is a technological solution to the conceptual proposal of preventive and proactive detection strategy. This factor not only helped create consensus between the federal and state governments, but also harnessed PHC-level support.

“*I hope to implement and disseminate this model, and that it truly becomes a pillar of change at a state level, and then at a national level*.” (Interview – PHC Physician)

#### Technological resource availability

We observed that the availability of technological resources facilitated the implementation of technology-based NCD tools and solutions. In this case, complete technological resource availability was a requirement for entering into the Casalud model and the Networks of Excellence; if PHCs did not have a particular piece of equipment, the FCS supplied it. As to be expected, we detected sustained improvement in PHC technology infrastructure where Casalud was implemented. The main improvements were observed in computer availability and internet connectivity in doctors’ offices: between 2013 and 2015 the number of available computers in all PHCs studied rose from 37 to 44 in one state and from 46 to 62 in the other. Similarly, the percentage of doctors’ offices with internet access increased from 0% in 2013 to 78% in 2015 in one state, and from 31% to 100% in the other.

Although we did observe these important improvements, once the FCS made the technological resources and medical equipment available, PHCs, local healthcare departments and state authorities were responsible for replenishing supplies. For example, PHCs and local healthcare department authorities must coordinate their procurement activities to assure a full stock of glucose testing strips for the MIDO system. Additionally, the FCS could not guarantee the installation and upkeep of computers at PHCs, nor the provision of continual internet access. These issues may affect the long-term viability of the model, as local authorities are subject to their own budget and planning constraints, and may suffer from a lack of information technology support. However, we conclude that, overall, the availability of these technological resources eased implementation of the model.

#### Technical adaptation and support of the model’s tools

A crucial part of the intervention implemented by the FCS was the continuous improvement of the tools it deployed to the field. Since the Casalud model relies on technological tools, the support and adaptation of these instruments is particularly relevant in the model’s implementation process.

The most important recent updates were that the SI-MIDO software, SIC, and AbastoNET were all standardized to match Ministry of Health requirements, catalogues and variables. Additionally for SIC, new variables regarding personal data, diagnosis and medical consultations were added, and the synchronization process was optimized. The Digital Portfolio is now multi-channel (web-based, Android app and offline through a USB), and includes a scientific kiosk with peer-reviewed articles. New methods were added to AbastoNET to notify stockouts daily, and the report design and development were made more intuitive. Finally, the structure of the video-lectures contained in PIEENSO was changed, and new video-lectures on prevention of NCDs, prescription of lifestyle changes and new pharmacologic management strategies were added. Detailed information on software updates can be found in Additional file 1.

As an illustrative example, the updating process of the PIEENSO platform took user experiences and comments into account through a straightforward opinion poll with feedback. This led HCPs to receive this process positively, which in turn became a key enabler for the PIEENSO tool.

Despite the fact that technology and associated improvements are considered an enabler, we observed a perceived lack of systematization in the process of improving the technological innovations. This deficiency was observed in the assessment of specific PHC needs during the adoption of these tools, the updating process, and the delivery of technological support services by the FCS and the Ministry of Health. In contrast to the positive PIEENSO upgrade process mentioned just above, less positive experiences were reported with the MIDO Backpack tablets, SIC and AbastoNET upgrading processes.

Interviewees referenced an episode in which MIDO Backpacks were collected by the FCS in order for the tablets to be updated, but the FCS never defined a clear schedule for this process. When time passed and the Backpacks had not been returned to the PHCs, this uncertainty gave way to a sense of general distrust about the adaptation and updating process of other tools. A similar situation occurred with the updating of SIC, referenced in the Administrative Practices section of the description of Inhibitors.

These examples of the updating process underscore the need for constant communication between service providers, policymakers and PHC personnel. The improvement process may be facilitated to a greater degree with the presence of more effective communication, both top-down and bottom-up. By improving downward and horizontal communication flows during the continuous improvement process, healthcare programs and interventions can increase the chances of having a successful implementation process and to build trust among those ultimately responsible for the implementation of a healthcare innovation: the physicians and PHC personnel. Clear schedules for updates might also improve the adaptation and support process, enhancing the enabling role of this factor.

#### Capacity-building strategies and mechanisms

The Online Certificate is perceived as a major enabler for the implementation of the Casalud model. Through it, HCPs are updated on recent advances in NCD detection and treatment and introduced to Casalud’s conceptual innovations, thus becoming more familiar with the model. As of May 2016, three rounds of HCPs had graduated from the Online Certificate program (224 individuals in the two studied states).

Independent assessment of HCP knowledge improvements showed that the Online Certificate improved knowledge, especially regarding the types of tests used for detecting NCDs, and goals and control levels for NCD biometrics. Social workers and nurses were the HCPs who registered the greatest increase in total NCD knowledge (30% and 14% improvement, respectively, measured with anonymous survey questions related to NCD knowledge).

In general, perception of the tool was positive:

“*The Certificate has given personnel better tools and a necessary update on NCD-related topics*.” (Interview – PHC director)

Of the HCP participants in both states, 94% and 81% reported being satisfied or very satisfied with the Online Certificate. In general, participants agreed that the Online Certificate taught them new things (89%) and that this knowledge was useful for their work at PHCs (87%). The capacity-building enabler was strengthened the most through this pillar of Casalud, which helped to familiarize HCPs with the tools being implemented at PHCs; 90% of participants in the Online Certificate reported being familiar with all of Casalud’s technologies.

### Inhibitors

#### Administrative practices

Administrative practices were perceived as one of the most relevant inhibitors to the model’s implementation, with entrenched administrative practices affecting the adoption of new technologies. Effecting changes in current administrative practices is a slow process, especially taking into account that administrative personnel, on average, report spending 10.5 years working in the health system.

The implementation of the SIC software involves the transition from paper-based records to digital records, which is difficult in part due to the onerous task of changing existing system regulation. As changes to normative frameworks take time to occur, the lack of an explicit federal instruction to stop using paper-based records was a relevant inhibitor to the model. Healthcare providers had to duplicate their work by entering the information in both the paper-based and the digital system. Observations showed that physicians and nurses filled out, on average, six different medical record formats per patient. In particular, there was a continued use of paper-based medical records, explained partially by current legislation that requires PHCs to produce hand-written reports for government officials. We found that 41% of the physical formats that were filled out during observations were requirements for the local healthcare jurisdiction and/or state authorities, 19% of paperwork was used for internal measurement of employee productivity, and the remaining 40% corresponded to federal level epidemiological measurements not directly used for PHC healthcare decision-making. These paperwork requirements act to the detriment of the model’s digital alternative: the SIC system.

SIC was designed to be a hybrid system, capable of operating both online and offline to allow for the intermittent availability of internet in PHCs. However, technical problems arose during the synchronization of data from the offline to online databases, resulting in PHC personnel believing that information had been lost. This, and other situations, created distrust and thus an incentive for PHC personnel to continue to fill out paper-based records as a backup. This means that physicians and HCPs often duplicated their work; for example, patients detected by MIDO were registered both digitally and manually, creating a double record, one online and one paper-based. A PHC director stated:

“*SIC is inoperative, as it cannot be updated in every office. There is no use in capturing and keeping data up-to-date if, in the end, physicians are not able to use the information. This is why we have to keep using paper files*.” (Interview)

The SIC system was systematically underused, because the personnel at the PHCs considered that infrastructural constraints made the uploading and synchronizing process between computers too difficult. As a PHC director affirmed:

“*I registered* [the patients treated by the director], *but I couldn’t see the registries in any other computer, even if they had the program installed*.” (Interview)

#### PHC human resources

**Fig. 1:**
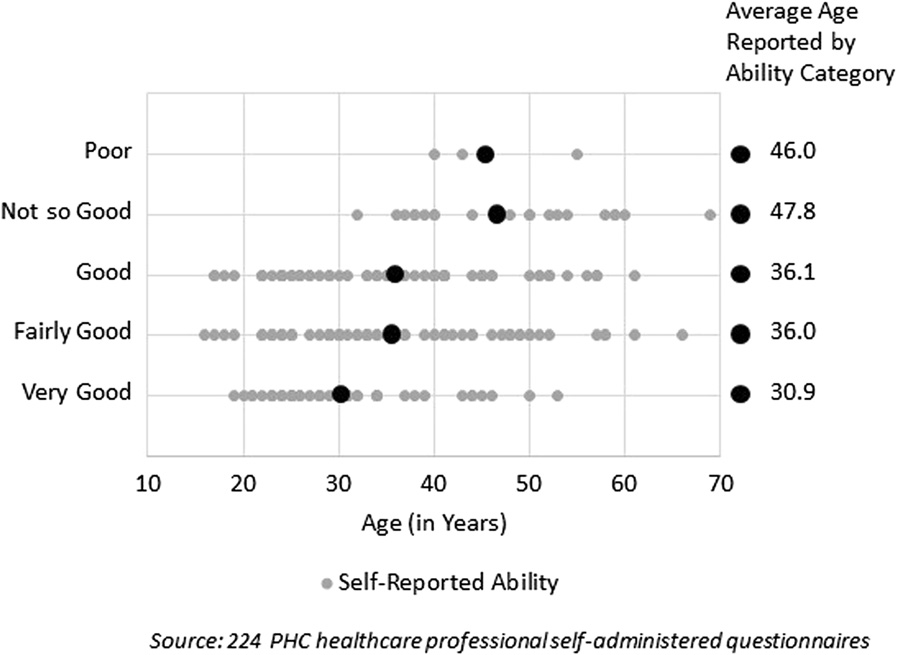
Self-reported ability to use a computer

PHC personnel show knowledge gaps in their ability to operate new technologies (Fig. 1). PHC personnel were asked how they rated their ability to use a computer; a clear link was observed between age and ability category (measured with an ANOVA). Younger PHC employees consistently report higher skill levels than older workers.

Another relevant factor is related to high human resource turnover rates within PHCs (Table 3). Structural characteristics of the Mexican healthcare system of medical training require nursing and medical school students, interns and residents to rotate through various environments for short periods of time. In the majority of PHCs studied, these students, interns and residents were the ones who operated the MIDO Mobile Cart, updated SIC or participated in Casalud capacity-building programs. Long-term, ongoing and comprehensive training of HCPs is difficult in a human resource setting limited by a high turnover rate.

**Table 3:** Personnel turnover rates

Position	Turnover rate 2014–2015 (Percentage reporting in 2015 not having been at that PHC in 2014)
PHC directors	33.3%
Physicians	26.0%
Nurses	38.9%
Other health provider	63.2%
Social worker	47.6%
Other (Operative)	44.4%
Other (Administrative)	20.0%

Source: 224 PHC healthcare professional self-administered questionnaires

In many observed cases, the human resource turnover caused a constant reorganization of functions, making it difficult for HCPs to specialize in the particular requirements of the Casalud model.

As a PHC director said:

“*My intern did the detections, but then a physician retired and the PHC administrative assistant couldn’t find someone to cover his place, so I had to send the intern to an office and I stayed with the cart, but it’s very hard. I have many other things to do: it is not possible to spend all my time on MIDO and SIC*.”

Finally, it is important to mention the fact that PHCs must manage multiple health programs and diverse patient profiles simultaneously. A limited number of HCPs treat conditions as diverse as pregnancies, chronic diseases, vaccinations and paediatric diseases, and given human resource constraints, this diversity can be difficult to manage. This, added to the personnel turnover rates, inhibits the implementation of the Casalud model.

#### Shared vision on the model’s validity and use

There were at least three elements that hindered the development of a shared vision for the Casalud model’s implementation.

The first element was in relation to street-level bureaucratic decisions, defined as decisions made by public workers who interact with beneficiaries on behalf of the state. We use the framework defined by Lipsky [33], who explains how street level bureaucrats abandon “*abstract goals to mitigate the immediate and persistent pressures of their working environment*” [34]. These pressures included fluctuating workloads, unclear leadership on specific programs, and provider-patient relationships. Personal relationships, especially in healthcare, can have a strong impact on patients’ ongoing decisions to seek medical attention with a certain provider [35]. In this study, a number of interviewed patients stated that they either changed or were willing to change PHC due to personal problems with physicians, nurses or administrative staff. This shows that factors that stand formally outside Casalud’s implementation, such as personal relationships, might influence the effectiveness of the implementation process. This is compounded by the fact that such issues might not be easily assessed a priori.

The second element that might have affected the development of a shared vision for the Casalud model’s implementation was the complex local and regional framework of the Mexican Ministry of Health within which it was operating. Federal, state and municipal officials, as well as PHC directors, have (either direct or indirect) influence on, and discretion over Casalud’s implementation. As a result, there may be a number of different perceptions, approaches and mandates regarding the model present at the same time. While at a system level political support was key to enabling the implementation of the model, at an institutional level, political situations sometimes inhibited the construction of a shared vision about it. A lack of clear top-down information flow, compounded by ineffective communication strategies, impeded the construction of a homogeneous shared vision of the model.

The third element was the divergence in opinions across PHCs towards Casalud’s components and tools. While this might have been caused by the street-level bureaucratic decisions mentioned above, we cannot rule out the possibility that it might have been caused by lack of uniformity in communication strategies surrounding the implementation process. The effective adoption of technologies goes beyond the tool itself and requires an explicit framework, a narrative of why it is useful, and constant mentoring and supervision of the operation. The human processes are more important than the technological tool. Divergent opinions on the MIDO Backpack help illustrate this point:

“*We need the tablets* [from MIDO Backpack] *back, working properly, because their use is practical. I was used to carrying out detections with that technology and it was very helpful.*” – PHC physician (Interview)

“*We never used it* [the MIDO Backpack]. *It affected the PHCs who used it, but for us the MIDO Backpack is non-existent*.” – PHC director (Interview)

The PIEENSO platform was designed when first implementing Casalud in order to assist with the construction of a shared vision, and it was very well received. Additional training on each tool, especially on SIC and MIDO, also proved helpful during the implementation process of each pillar. However, the lack of a cross-cutting communication strategy and/or an explicit strategy for navigating the complex health system framework proved to be an inhibitor when attempting to modify street-level bureaucratic cultures.

## Conclusions

**Fig. 2:**
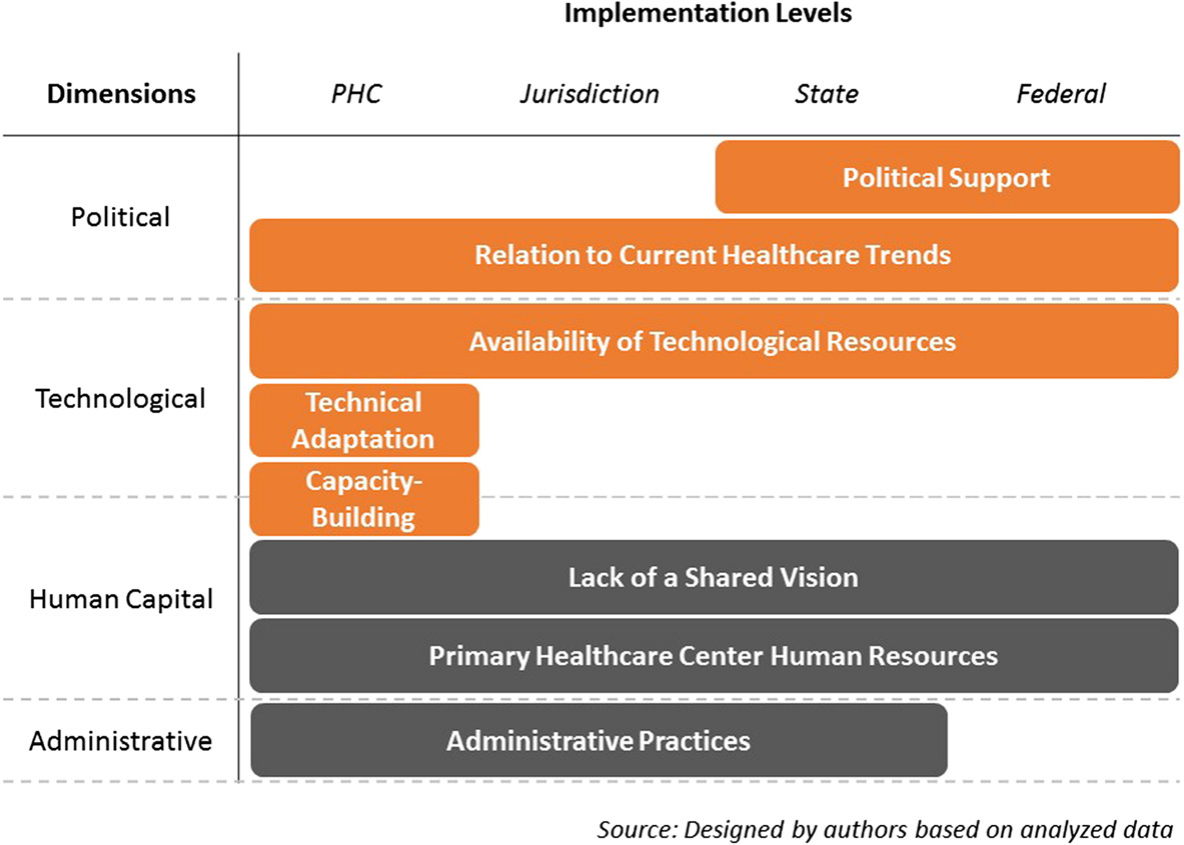
Implementation levels and dimensions

The most pressing concern in need of investigation was the identification and mapping of key bottlenecks. This is why, after coding findings, categories were organized depending on the implementation level (PHC, local healthcare department, state, or federal) as well as by their implementation dimension (political, technological, administrative or human resources). We framed our findings according to the following diagram (Fig. 2), where orange corresponds to enablers and grey to inhibitors.

We found that enablers and inhibitors are located across all implementation levels and dimensions, although Fig. 2 clearly shows how enablers are associated with ‘political’ and ‘technology’ dimensions. Technical adaptation and support of the model’s tools and the capacity-building strategies and mechanisms were found solely at the PHC level. ‘Capacity-building’ is the only enabler that successfully straddles two dimensions: human capital and technology. This is especially relevant, as it might become a strategy for overcoming human resource concerns regarding technological literacy and skills. We highlight the role political support played in scaling up an innovative, patient-centred model, based on current policy trends. These political enablers, which operate at the federal level, reinforced the PHC level enablers (constant adaptation of the model’s tools to meet HCPs’ needs, and capacity-building strategies that improved NCD knowledge and treatment through Casalud’s tools). The enablers show that it is possible to induce change in rigid healthcare systems, as well as improve HCP knowledge, through innovation and technology. When paired with strong support from key authorities, innovative models are likely to be scaled-up quickly and completely.

These dimensions interact in a fluid manner. Political support for the model caused rapid adoption of technologically valid tools, and although the Casalud tools disrupted PHC-level entrenched administrative practices, they were not able to totally replace these practices. Low technological literacy can be an inhibitor, especially for the MIDO Mi Salud and MIDO Mi Diabetes applications, although it is relevant to note that this situation is not unique to Casalud. Several policy studies have shown that technology-intensive health interventions, such as mHealth programs, share this common barrier [36, 37]. Low technological literacy is a major inhibitor because most of Casalud’s tools rely on new technology.

The specificities and complexities of the administrative practices and human resource constraints required a constant adaptation of the model’s tools (in particular, the MIDO Mobile Cart and the MIDO Backpack). If the updating process (and indeed the entire model) is not communicated in an efficient way to PHC personnel, they can develop a mistrust of the adaptation process and potentially reject some of the tools. Furthermore, PHC-specific management practices can either enable or hinder implementation of the model. As Casalud requires physicians and other HCPs to modify the way they interact with their patients – the basis of their day-to-day work – HCPs’ perceptions of the validity, use and reach of the model’s tools are crucial. Thus, efficient communication methods are a vital ingredient in the construction of a shared vision around Casalud.

The implementation process of the Casalud model shows that technology-based integrated healthcare models may encounter important barriers in some PHCs with entrenched practices. These blockages might not be readily apparent at a system-wide or federal level, and might only become visible at a PHC level, where legislation, healthcare culture and street-level decisions mix with state level rules and practices and system-wide human and technological resource constraints. Some of the problems arising might not be foreseeable, as they involve cultures specific to individual PHCs or healthcare professionals, whereas other barriers can be identified more easily before the implementation process begins.

The challenges faced by Casalud are not unique to Casalud alone; we present our three main recommendations here so they may be useful to other technology-based healthcare models.

The first recommendation is that all technological tools must be constantly adapted and that maintenance service must be not only efficient, but also user-centred. PHC street-level bureaucrats are the ones actually implementing this public policy on the frontlines, and they possess considerable autonomy and discretion. If a technology does not fit into their routine workplace activities, they may choose not to implement the particular tools of that technology.

We recommend that implementing technological health innovations involves generating a shared vision regarding the model. This requires clear communication between PHC personnel, technicians in charge of maintenance and update of the model’s tools, experts designing and improving the model’s logic, and decision-makers escalating the pace of implementation. These communication strategies should be open, both bottom-up and top-down, in order to promote the joint construction of a vision around the model, its components and tools.

Finally, we recommend that technology-based comprehensive interventions be open to change and adaptation. Many of the barriers Casalud encountered while being implemented were not easily foreseeable and were too specific to be comprehended without a complex and time- and resource-consuming analysis. While in-depth analysis might be desirable in theory, the reality is that the window of opportunity for the large-scale implementation of an ambitious, innovative intervention might not be open for long. If the system-level facilitators are in place, a model can be implemented, as long as openness toward adaptation of its tools and components is maintained.

Closely related to this recommendation, we found that Casalud is a model in a constant process of adaptation, and throughout its implementation, it has shown a remarkable ability to adapt to potentially adverse circumstances. By creating strategies to build a shared vision and gather key support from relevant stakeholders at state and PHC levels, Casalud will continually improve its implementation process, becoming a key factor in the overall improvement of the Mexican healthcare system. Casalud may help other countries design nation-wide comprehensive NCD programs to help them reduce the burden of chronic diseases and attain their country’s Sustainable Development Goals.

## Abbreviations

FCS, Carlos Slim Foundation; HCP, healthcare professional; Networks of Excellence, Networks of Excellence of the National Strategy for the Prevention and Control of Pre-obesity, Obesity and Diabetes; NCD, Non-Communicable Disease; PHC, primary health clinic; T2D, type 2 diabetes mellitus

## Additional file

Additional file 1: Software programs and upgrades. (DOC 171 kb)
