# Supplementary material for: Enablers and inhibitors of the implementation of the Casalud Model, a Mexican innovative healthcare model for non-communicable disease prevention and control
Source: Health Res Policy Syst. 2016 Jul 22;14:52. doi: 10.1186/s12961-016-0125-0 (PMC4957422; doi:10.1186/s12961-016-0125-0)
Supplement: Supplementary file 1 [file 12961_2016_125_MOESM1_ESM.doc]

Annex 1: Software programs and upgrades

Associated Tool	Description of the Software	Upgrade
MIDO Module and MIDO Backpack	Proprietary software SI-MIDO systematizes NCD screening by registering personal data, calculating NCD risk from a questionnaire, and storing the values of the screening tests. The system works online and offline for the uploading and storing of data.	SI-MIDO is in Version 3.0. The most important update was the adaptation of the risk assessment questionnaire that SI-MIDO uses to match the questionnaire required by the Ministry of Health (called the Risk Factors Questionnaire). Additionally, SI-MIDO links with SIC to confirm diagnosis and follow up with patients. The synchronization process and communication with the MIDO Android App were optimized.
Chronic Information System (SIC)	Proprietary software SIC contains variables important for people living with NCDs, including sociodemographic data, NCD diagnosis and medical office visits. The system works online and offline for the uploading and storing of data.	Currently in Version 3.0. The most important changes were: the standardization of catalogues and variables to match those of Mexico's Ministry of Health; the addition of new variables regarding personal data, diagnosis and medical consultations; an optimized synchronization process; and the ability to run new reports with robust business intelligence variables.
Digital Portfolio	Digital platform of tools to enable health professionals to improve prevention and disease management. It includes a scientific kiosk, clinical guidelines, health calculators, vademecum and massive online open courses.	Currently in Version 3.0. Major changes occurred that now allow the digital portfolio to be multi-channel (web-based, Android app and offline through a USB). Additionally, a scientific kiosk was developed (with articles from the *New England Journal of Medicine*) and Mexico's National Academy of Medicine, calculators were upgraded to include games, and the interface was changed to a new HTML5 graphic interface.
AbastoNET	An online information system that standardizes metrics for stock management at PHCs.	Currently in Version 2.0. The principal upgrade was the standardization of the catalogue of medicines to match those of the Council of General Health of the Ministry of Health. Additionally, new methods were added to notify of stock-outs, and report design and development was made more intuitive.
PIEENSO	PIEENSO is based on a robust educational platform that offers asynchronous education through tailored online courses for HCPs, with traceability for any student enrolled in the platform.	PIEENSO is currently in Version 2.0. The most important changes were the structure of the video-lectures and the addition of new video-lectures regarding prevention of NCDs, prescription of lifestyle changes, and new pharmacologic management strategies.

Source: Fundación Carlos Slim (2015)
